# Broad-spectrum neuroprotection exerted by DDD-028 in a mouse model of chemotherapy-induced neuropathy

**DOI:** 10.1097/j.pain.0000000000002963

**Published:** 2023-08-07

**Authors:** Elena Lucarini, Laura Micheli, Raghavan Rajagopalan, Clara Ciampi, Jacopo J.V. Branca, Alessandra Pacini, Massimo Leandri, Parthasarathi Rajagopalan, Carla Ghelardini, Lorenzo Di Cesare Mannelli

**Affiliations:** a Department of Neuroscience, Psychology, Drug Research and Child Health (NEUROFARBA), Pharmacology and Toxicology Section, University of Florence, Florence, Italy; b Daya Drug Discoveries, Inc, St. Louis, MO, United States; c Department of Experimental and Clinical Medicine, Anatomy and Histology Section, University of Florence, Florence, Italy; d Department of Neuroscience, Rehabilitation, Ophthalmology, Genetics, Maternal and Child Health, University of Genoa, Genoa, Italy

**Keywords:** DDD-028, Neuroprotection, Nerve conduction, Neurofilament, Paclitaxel, Intraepidermal nerve fiber

## Abstract

Neurotoxicity of chemotherapeutics involves peculiar alterations in the structure and function, including abnormal nerve signal transmission, of both the peripheral and central nervous system. The lack of effective pharmacological approaches to prevent chemotherapy-induced neurotoxicity necessitates the identification of innovative therapies. Recent evidence suggests that repeated treatment with the pentacyclic pyridoindole derivative DDD-028 can exert both pain-relieving and glial modulatory effects in mice with paclitaxel-induced neuropathy. This work is aimed at assessing whether DDD-028 is a disease-modifying agent by protecting the peripheral nervous tissues from chemotherapy-induced damage. Neuropathy was induced in animals by paclitaxel injection (2.0 mg kg^−1^ i.p). DDD-028 (10 mg kg^−1^) and the reference drug, pregabalin (30 mg kg^−1^), were administered per os daily starting concomitantly with the first injection of paclitaxel and continuing 10 days after the end of paclitaxel treatment. The behavioural tests confirmed the antihyperalgesic efficacy of DDD-028 on paclitaxel-induced neuropathic pain. Furthermore, the electrophysiological analysis revealed the capacity of DDD-028 to restore near-normal sensory nerve conduction in paclitaxel-treated animals. Histopathology evidence indicated that DDD-028 was able to counteract effectively paclitaxel-induced peripheral neurotoxicity by protecting against the loss of intraepidermal nerve fibers, restoring physiological levels of neurofilament in nerve tissue and plasma, and preventing morphological alterations occurring in the sciatic nerves and dorsal root ganglia. Overall, DDD-028 is more effective than pregabalin in preventing chemotherapy-induced neurotoxicity. Thus, based on its potent antihyperalgesic and neuroprotective efficacy, DDD-028 seems to be a viable prophylactic medication to limit the development of neuropathies consequent to chemotherapy.

## Abstract 2

DDD-028 counteracts the establishment of neuropathic pain and sensory nerve conduction disturbance induced by paclitaxel, reducing the morphological and morphometric damage to the peripheral nervous system.

## 1. Introduction

The development of neuropathy is the predominant reason for dose modification and discontinuation of some chemotherapeutics, such as taxanes, thereby adversely affecting cancer treatments.^20,63^ Paclitaxel, a plant-derived antineoplastic agent belonging to the family of taxanes, is one of the most effective drug against solid tumors.^78^ Unfortunately, the dose and the duration of the treatments with paclitaxel positively correlate with the establishment of neuropathy that can persist for months or years after the end of the treatment.^12,44,82^ Despite decades of pharmacological research, no agent has been shown to successfully prevent chemotherapy-induced neuropathy (CIN). In 2014, and in the updated version published in 2020, the American Society of Clinical Oncology (ASCO) produced guidelines which extensively addressed the prevention and management of CIN. After a systematic review, ASCO guidelines concluded that no neuroprotective agent can be addressed with confidence as a neuroprotective strategy against CIN in the general setting.^38,56^ Under the European Society for Medical Oncology 2018 guideline for management of cancer pain in adult patients, pregabalin and gabapentin along with duloxetine and tricyclic antidepressants were the drug most recommended for neuropathic pain first-line treatment.^32^ Paclitaxel leads to both macrophage infiltration in the dorsal root ganglia (DRG) and is responsible for microglia or astrocyte activation in the spinal cord^71^ which cause a prolonged activity in dorsal horn neurons.^19^ Although the functional impairment associated with CIN often reflects a large-fiber polyneuropathy, a damage to small and thinly myelinated (Aδ) and unmyelinated (C) fibers can also occur, resulting in neuropathic pain and autonomic dysfunction. To assess the presence of small-fiber neuropathies by nerve conduction studies remains a challenge.^31^ So, the diagnosis in patients is based on quantitative sensory testing and on density assessment of intraepidermal nerve fibers (IENFs)^26^ and bare nerve endings of Aδ and C fibers which transmit noxious mechanical and thermal information.^65^ It has been observed that paclitaxel treatment causes a loss of IENFs,^85^ which has been associated with chronic painful neuropathic conditions, arising from diabetes, complex regional pain syndrome, postherpetic neuralgia, and other ailments,^42,67,72,84^ strengthening the close link between this phenomenon and chronic pain.^13^ Paclitaxel also induces significant and dose-dependent alterations in the nerve conduction properties in animals^12,16,54^ and humans.^5,41,73^ Pain was reported to have a high incidence in neuropathic patients showing abnormalities in nerve conduction studies, although these tests only provide information on impairment of Aβ fibers.^31,33^

Commonly used medications, such as gabapentin, lamotrigine, or pyridoxine plus pyridostigmine, showed low efficacy against neuropathic pain in random clinical trials.^55,62,89^ Previously, several attempts have been performed to treat or prevent CIN with various neuroprotective drugs, but most of them are either ineffective or cause neurological side effects.^25,89^ Thus, novel therapeutic strategies to prevent chemotherapy-induced damage to the peripheral nervous system effectively and safely are in critical need. Our previous studies highlighted the therapeutic potential of the pentacyclic pyridoindole heterocycle DDD-028 in a rat model of paclitaxel-induced neuropathy.^61,75,76^ DDD-028 showed a good antihyperalgesic efficacy mediated by the modulation of nicotinic system, particularly by the α7 nAChR subtype.^61^ Moreover, DDD-028 displayed disease-modifying properties by counteracting the damage caused by chemotherapeutic treatment to both the central and peripheral nervous system.^61^

The aim of the present work was to further investigate the neuroprotective efficacy of the repeated administrations of DDD-028 in paclitaxel-treated mice, with particular attention to the pathophysiology of the peripheral nervous system. Accordingly, electrophysiological evaluation of nerve conduction was conducted concurrent to histological and molecular assessment of sciatic nerves and DRG.

## 2. Methods

### 2.1. Animals

For all the experiments described below, male CD-1 mice (Envigo, Varese) weighing approximately 20 to 25 g at the beginning of the experimental procedure were used. Animals were housed in Ce.S.A.L. (Centro Stabulazione Animali da Laboratorio, University of Florence) and used at least 1 week after their delivery. Ten mice were housed per cage (size 26 × 41 cm^2^); animals were fed with standard laboratory diet and tap water ad libitum, kept at 23 ± 1°C with a 12-hour light–dark cycle, light at 7 am. All animal manipulations were conducted according to the Directive 2010/63/EU of the European Parliament and of the European Union council (22 September 2010) on the protection of animals used for scientific purposes. The ethical policy of the University of Florence complies with the Guide for the Care and Use of Laboratory Animals of the U.S. National Institutes of Health (NIH Publication No. 85-23, revised 1996; University of Florence assurance number: A5278-01). Formal approval to conduct the experiments described was obtained from the Italian Ministry of Health (No. 498/2017-PR) and from the Animal Subjects Review Board of the University of Florence. Experiments involving animals have been reported according to ARRIVE guidelines.^60^ All efforts were made to minimize animal suffering and to reduce the number of animals used.

### 2.2. Paclitaxel mouse model of neuropathy and DDD-028 administration

Mice treated with paclitaxel (2.0 mg kg^−1^) were injected i.p. on 4 alternate days (days 1, 3, 5, and 8).^59^ Paclitaxel was dissolved in a mixture of 10% saline solution and Cremophor EL, a derivative of castor oil and ethylene oxide that is clinically used as the paclitaxel vehicle. DDD-028 was suspended in 1% carboxymethylcellulose and administered per os daily at 10 mg kg^−1^ dose^61^ to induce a protective effect against paclitaxel-induced neuropathy. DDD-028 administrations were performed from the beginning of paclitaxel administration to the end of the experiment. The reference drug pregabalin (30 mg kg^−1^)^1,81^ was suspended in 1% carboxymethylcellulose and administered per os daily following the same protocol for DDD-028 treatment. Control animals were treated with the vehicles.

### 2.3. von Frey test

The animals were placed in 20 × 20-cm plexiglass boxes equipped with a metallic meshy floor, 20 cm above the bench. Habituation of 15 minutes was allowed before the test. An electronic von Frey hair unit (Ugo Basile, Varese) was used: the withdrawal threshold was evaluated by applying a force ranging from 0 to 5.0 g with a 0.2-g accuracy. A punctuate stimulus was directed at the midplantar area of each anterior paw from below the meshy floor with a plastic tip and the withdrawal threshold was automatically displayed on the screen. The paw sensitivity threshold was defined as the minimum pressure required to elicit a robust and immediate withdrawal reflex of the paw. Voluntary movements associated with locomotion were not taken as a withdrawal response. Stimuli were applied on each anterior paw at an interval of 5 seconds. The measure was repeated 5 times and the final value was obtained by averaging the 5 measures.^80^

### 2.4. Cold-plate test

Thermal allodynia was assessed using the cold-plate test. With minimal animal–handler interaction, mice were taken from home cages and placed onto the surface of the cold plate (Ugo Basile, Varese) maintained at a constant temperature of 4°C ± 1°C. Ambulation was restricted by a cylindrical plexiglass chamber (diameter: 10 cm, height: 15 cm), with open top. A timer controlled by the foot pedal started computing the response latency from the moment the mouse was placed onto the cold plate. The pain-related behaviour (licking of the hind paw) was observed, and the time (seconds) of the first appearance was recorded. The cutoff time of paw lifting or licking was set at 30 seconds as previously reported.^7^

### 2.5. Paw-pressure test

Mechanical hyperalgesia was determined by measuring the latency in seconds of paw withdrawal from a constant mechanical pressure (applied to its dorsal surface).^79^ A 15-g calibrated glass cylindrical rod (diameter = 10 mm) chamfered to a conical point (diameter = 3 mm) was used to exert the mechanical force. The weight was suspended vertically between 2 rings attached to a stand and was free to move vertically. A single measure was made per animal. A cutoff time of 40 seconds was used.

### 2.6. Assessment of nerve conduction in vivo

The evaluation of sensory nerve conduction was performed in vivo at the level of the tail and the hind limbs according to the methods previously reported in the literature.^54,91^ All the measures were performed on the animals under light anesthesia (2% isoflurane). Nerve stimulation was performed through 2 subdermal needle electrodes (length 10 mm, diameter 0.35 mm, placed 5 mm apart from each other) inserted at the distal end of the tail (caudal nerve) or at the level of the fourth finger of the hind limb (sural nerve), using electrical impulses of intensity 10 mA (supramaximal) and duration 0.05 (caudal nerve) or 0.2 milliseconds (sural nerve), delivered at the rate of 1/s. Signals were recorded by 2 subdermal needle electrodes (length 10 mm, diameter 0.35 mm, placed 5 mm apart from each other) inserted proximal to the stimulating electrodes at a distance of 20 to 40 mm in the tail and 10 to 30 mm in the hind limb. The ground electrode was positioned under the skin at an intermediate distance between the stimulus and recording electrodes. For the assessment of motor nerve conduction, the positions of recording and stimulation electrodes were reversed, maintaining the same distances, and using an intensity of 10 mA (supramaximal), delivered at a rate of 1/s. Thirty responses for sensory conduction and 10 responses for motor conduction were averaged to increase the signal-to-noise ratio. The nerve conduction velocity and the amplitude of the nerve response were calculated. The trigger signal was provided through a stimulus isolator (Stimulus Isolator, ADInstruments, Colorado Springs, CO). Signals from the electrodes were fed into a data acquisition system, which provided amplification with an overall gain of 20,000 for the nerve recordings and 3000 for motor responses. Signals were also filtered with a bandpass of 3 to 5000 Hz (Animal Bio Amp, ADInstruments) with digital sampling at 200 k/s (PowerLab 4/35, ADInstruments) and then processed offline with LabChart 8 (ADInstruments).

### 2.7. Immunohistochemistry and intraepidermal nerve fiber analysis

The mice hind limb paw skin was placed overnight at 4°C in 4% paraformaldehyde in PBS1X, transferred to 30% sucrose overnight, frozen, and cryosectioned at 50 µm sections transversally along the paw axis. Free-floating sections were incubated in PBS containing 0.3% Triton X-100 (TBS) 1 hour at room temperature and then in the primary antibody panaxonal marker PGP9.5 (ab108986, rabbit monoclonal [EPR4118] 1:600, Abcam) overnight at room temperature. Thereafter, the sections were rinsed with PBS1X and placed in a goat anti-rabbit IgG secondary antibody labelled with Alexa Fluor 488 (1:500) for 2 hours at room temperature in the dark. To stain nuclei, sections were incubated with DAPI in PBS for 10 minutes. After 3 washes in PBS and a final wash in distilled water, the slices were mounted using ProLong Gold (Life Technologies-Thermo Fisher Scientific, Milan) as a mounting medium. Digitalized images were collected at ×200 total magnification by a motorized Leica DM6000 B microscope equipped with a DFC350FX digital camera. Quantitative analysis of IENF density (fibers/mm) was performed by collecting 6 independent fields in the skin of each animal and counting the number of single PGP9.5-positive fibers crossing the epidermis–dermis boundary (basal membrane) by using the software ImageJ (NIH, Bethesda, MD). Secondary branching is excluded from quantification, according to the European Federation of Neurological Societies' guidelines.^52,58^

### 2.8. Neurofilament heavy-chain immunohistochemistry on the sciatic nerves and dorsal root ganglia

Formalin-fixed cryostat sections (7 μm) were washed 3 x phosphate-buffered saline (PBS1x) and incubated, at room temperature for 1 hour, in blocking solution (PBS1x, 0.3% Triton X-100, and 5% albumin bovine serum). The sections were subsequently incubated with primary antibody, anti-neurofilament heavy chain (NF-H sc-32729 mouse 1:100, Santa Cruz Biotechnology), overnight at 4°C. In the following day, the slides were washed with 3 × PBS1x and the sections were incubated in the dark with secondary antibody, goat anti-mouse IgG labeled with Alexa Fluor 488, in blocking solution at room temperature for 2 hours. After 3 × PBS1x wash for 10 minutes, the sections were incubated with DAPI, a nuclei marker, at room temperature for 5 minutes. Finally, the slides were mounted using Fluoromount (Life Technologies-Thermo scientific, Rockford, IL) as a mounting medium.

Negative control sections (no exposure to the primary antisera) were processed concurrently with the other sections for all immunohistochemical studies. Images were acquired using a motorized Leica DM6000 B microscope equipped with a DFC350FX camera (Leica, Mannheim). Immunofluorescent staining was measured as mean fluorescence intensity using ImageJ software (ImageJ, National Institute of Health, https://imagej.nih.gov/) by automatic thresholding algorithm and expressed in percentage in comparison to the vehicle + vehicle group set as 100%. Analyses were performed on at least 5 different images for each animal, collected through a ×40 objective.

### 2.9. Neurofilament light-chain evaluation in plasma

Blood was obtained by exsanguination from vena cava, and plasma levels of NF-L were analyzed by an ELISA assay according to the protocol provided by the manufacturer (Biomatik, USA, LCC).

### 2.10. Morphometric analysis

Sciatic nerves and DRG were fixed in 2.5% glutaraldehyde in cacodylate buffer pH 7.4 for 24 hours. The tissue samples were osmicated in a 1% solution of osmium tetroxide overnight under constant agitation. Before and after osmication, the tissues were repeatedly rinsed in 0.1 M sodium cacodylate at pH 7.4 and then embedded in epoxy resin. The tissues were cut into 0.8 μm sections using an ultramicrotome and each section was stained with toluidine blue.^35^ Counts and measurements were performed using image analyzing software (ImageJ 1.48). Micrographs were obtained using a Nikon Olympus BX40 and a 400X objective equipped with NIS F3.00 imaging software. For the sciatic nerve, myelin area, number of fibers (separated by axon diameter), and axon diameters were assessed. Alterations in DRG were analyzed by measuring the number of neurons showing multinucleolated nuclei as a sign of toxicity. Furthermore, the soma area was assessed by stratifying neurons in 3 sizes: small (<600 µm diameter), medium (>600 < 1200 µm diameter), and large (>1200 µm diameter).^27^ Analyses were performed on different images for each animal, collected through a X20 objective.

### 2.11. Statistical analysis

All the experimental procedures were performed by a researcher masked to the treatment. Results were expressed as mean ± S.E.M. of 6 to 8 animals per group from 2 experimental replicates. To calculate statistical significance of both behavioural and histological data, the analysis of variance was performed by one-way analysis of variance. A Bonferroni significant difference procedure was used as post hoc comparison. *P* values of less than 0.05, 0.01, or 0.001 were considered significant. Data were analyzed using the “Origin 9.1” software (OriginLab, Northampton, MA).

## 3. Results

### 3.1. Effect of DDD-028 on paclitaxel-induced neuropathic pain

**Figure 1.**
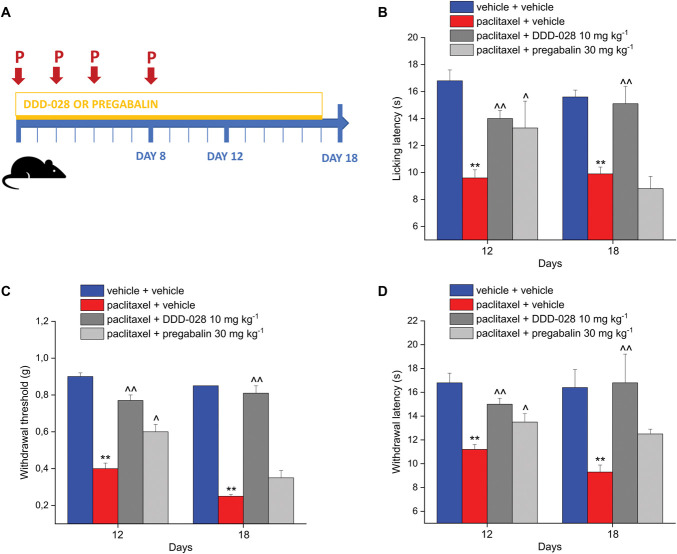
Effect of DDD-028 on paclitaxel-induced neuropathic pain*.* (A) Experimental scheme: paclitaxel 2.0 mg kg^−1^ was dissolved in a mixture of 10% Cremophor EL and saline solution and intraperitoneally (i.p.) administered on 4 alternate days (1, 3, 5, and 8). DDD-028 (10 mg kg^−1^) and pregabalin (30 mg kg^−1^) were suspended in 1% carboxymethylcellulose (CMC) sodium salt and daily per os administered starting on the first day of paclitaxel treatment and continuing 1 week after the end of treatment (from day 1 to day 17). The pain was measured in animals on days 12 and 18, 24 h after the last treatment. (B) Thermal allodynia was assessed by the cold-plate test, (C) mechanical allodynia was assessed by the von Frey test, and (D) mechanical hyperalgesia was assessed by the paw-pressure test. Each value represents the mean ± S.E.M of 6 to 8 mice performed in 2 different experimental sets. **P < 0.01 vs vehicle + vehicle; ^*P* < 0.05 and ^^*P* < 0.01 vs paclitaxel + vehicle.

Neuropathy was induced in animals by the injection of paclitaxel (2.0 mg kg^−1^ i.p). DDD-028 (10 mg kg^−1^) and the reference drug, pregabalin (30 mg kg^−1^), were administered daily per os starting on the first day of paclitaxel treatment and continuing for 10 days after the end of treatment (from day 1 to day 17). Pain was monitored in the animals in the course of the experiment, as reported in the scheme in Figure 1A. As previously demonstrated in rats,^61^ we confirmed that the repeated treatment with DDD-028 (10 mg kg^−1^, per os) counteracted the development of paclitaxel-induced neuropathic pain in mice. DDD-028 significantly increased the pain threshold of paclitaxel-injected mice at all the considered time points with no development of tolerance to the antihyperalgesic effect when the animals were placed on a cold surface (cold-plate test), whereas pregabalin's effect reached a statistical significance only on day 12 and was no more active on day 18 (Fig. 1B). The repeated administration of DDD-028 showed a similar efficacy in reducing paclitaxel-induced mechanical allodynia (von Frey test, Fig. 1C) and hyperalgesia (paw-pressure test, Fig. 1D). In paclitaxel-treated animals receiving DDD-028, both the withdrawal latency to a nonnoxious mechanical stimulus and the tolerance to a noxious mechanical stimulus were increased with respect to animals receiving the vehicle at each time point investigated, whereas pregabalin was active only on day 12 (Figs. 1C and D).

### 3.2. Effect of DDD-028 on nerve conduction impairments caused by paclitaxel administration

**Figure 2.**
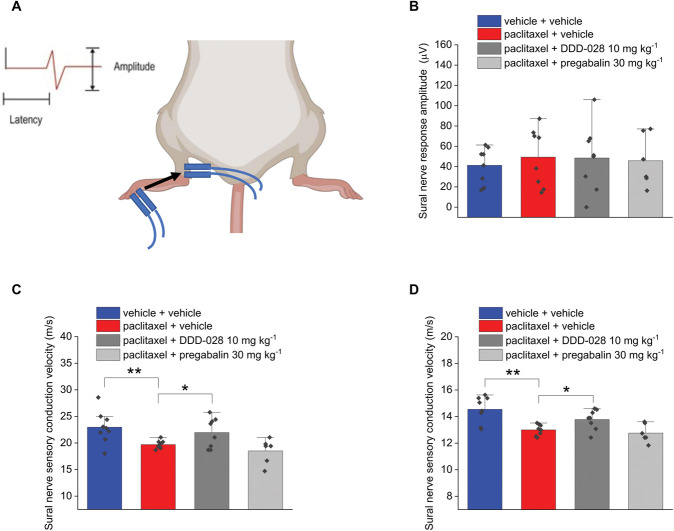
Effect of DDD-028 on sural nerve sensory conduction alterations induced by paclitaxel injection in mice. Paclitaxel 2.0 mg kg^−1^ was dissolved in a mixture of 10% Cremophor EL and saline solution and intraperitoneally (i.p.) administered on 4 alternate days (1, 3, 5, and 8). DDD-028 (10 mg kg^−1^) and pregabalin (30 mg kg^−1^) were suspended in 1% carboxymethylcellulose (CMC) sodium salt and daily administered per os starting on the first day of paclitaxel treatment and continuing 1 week after the end of treatment (from day 1 to day 17). Nerve conduction velocity was assessed on day 18, 24 h after the last treatment. (A) Scheme of the experimental setup, (B) amplitude of the sural nerve sensory response, and (C)–(D) sensory conduction velocity of sural nerve was calculated by considering both the latency to the response onset and the latency to the response peak. Thirty responses for sensory conduction were averaged to increase the signal-to-noise ratio. Each value represents the mean ± S.E.M of 6 to 8 mice performed in 2 different experimental sets. *P < 0.05 and **P < 0.01.

**Figure 3.**
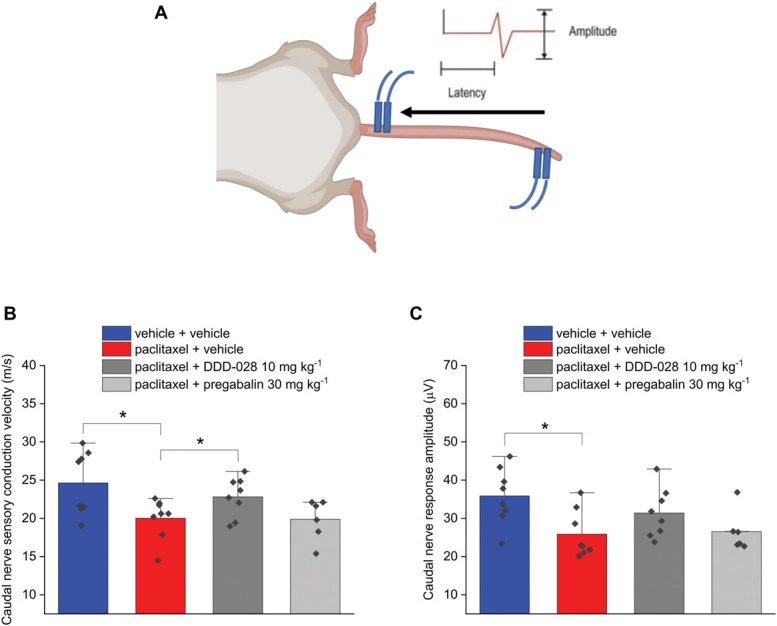
Effect of DDD-028 on caudal nerve sensory conduction alterations induced by paclitaxel injection in mice. Paclitaxel 2.0 mg kg^−1^ was dissolved in a mixture of 10% Cremophor EL and saline solution and intraperitoneally (i.p.) administered on 4 alternate days (1, 3, 5, and 8). DDD-028 (10 mg kg^−1^) and pregabalin (30 mg kg^−1^) were suspended in 1% carboxymethylcellulose (CMC) sodium salt and daily administered per os starting on the first day of paclitaxel treatment and continuing 1 week after the end of treatment (from day 1 to day 17). Nerve conduction velocity was assessed on day 18, 24 h after the last treatment. (A) Scheme of the experimental setup, (B) sensory conduction velocity of caudal nerve, and (C) amplitude of the caudal nerve sensory response. Thirty responses for sensory conduction were averaged to increase the signal-to-noise ratio. Each value represents the mean ± S.E.M of 6 to 8 mice performed in 2 different experimental sets. *P < 0.05.

**Figure 4.**
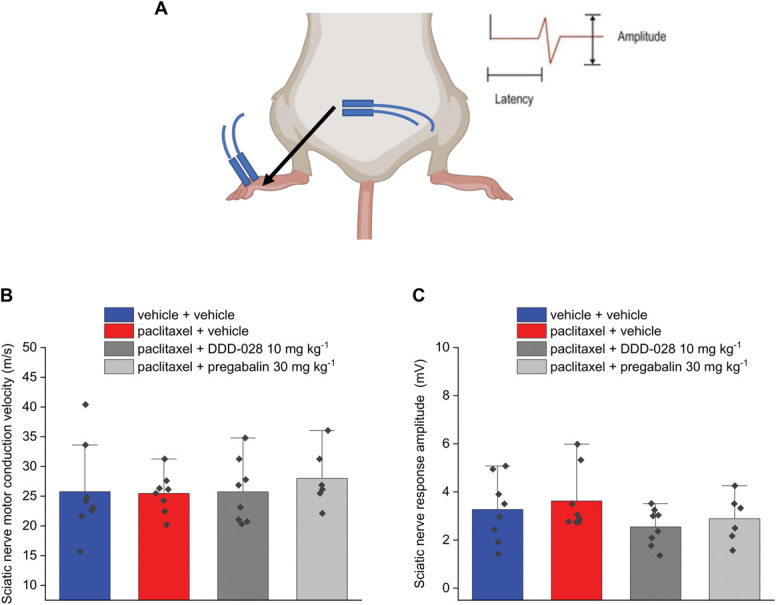
Effect of paclitaxel and DDD-028 on sciatic nerve motor conduction in mice*.* Paclitaxel 2.0 mg kg^−1^ was dissolved in a mixture of 10% Cremophor EL and saline solution and intraperitoneally (i.p.) administered on 4 alternate days (1, 3, 5, and 8). DDD-028 (10 mg kg^−1^) and pregabalin (30 mg kg^−1^) were suspended in 1% carboxymethylcellulose (CMC) sodium salt and daily administered per os starting on the first day of paclitaxel treatment and continuing 1 week after the end of treatment (from day 1 to day 17). Nerve conduction velocity was assessed on day 18, 24 h after the last treatment. (A) Scheme of the experimental setup, (B) motor conduction velocity of sciatic nerve, and (C) amplitude of the sciatic nerve motor response. Ten responses for motor conduction were averaged to increase the signal-to-noise ratio. Each value represents the mean ± S.E.M of 6 to 8 mice per group performed in 2 different experimental sets.

**Figure 5.**
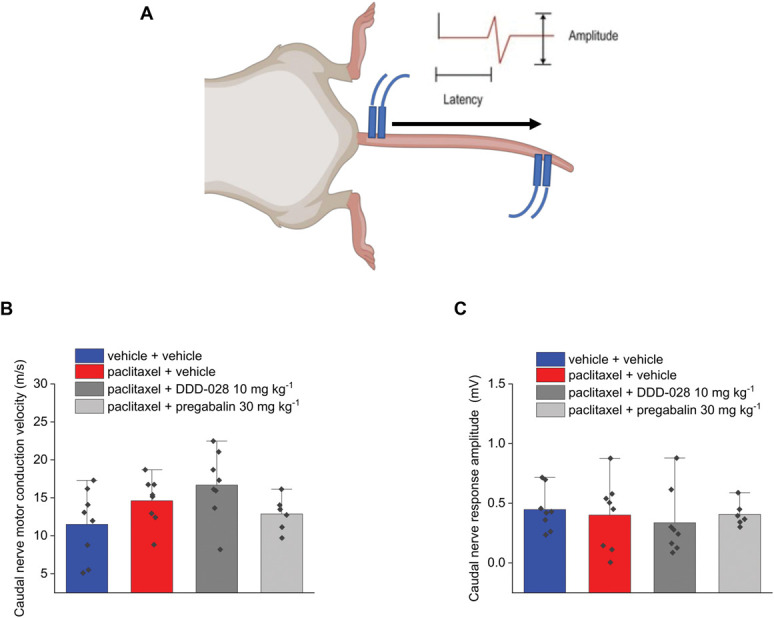
Effect of paclitaxel and DDD-028 on caudal nerve motor conduction in mice. Paclitaxel 2.0 mg kg^−1^ was dissolved in a mixture of 10% Cremophor EL and saline solution and intraperitoneally (i.p.) administered on 4 alternate days (1, 3, 5, and 8). DDD-028 (10 mg kg^−1^) and pregabalin (30 mg kg^−1^) were suspended in 1% carboxymethylcellulose (CMC) sodium salt and daily administered per os starting on the first day of paclitaxel treatment and continuing 1 week after the end of treatment (from day 1 to day 17). Nerve conduction velocity was assessed on day 18, 24 h after the last treatment. (A) Scheme of the experimental setup, (B) motor conduction velocity of caudal nerve, and (C) amplitude of the caudal nerve motor response. Ten responses for motor conduction were averaged to increase the signal-to-noise ratio. Each value represents the mean ± S.E.M of 6 to 8 mice per group performed in 2 different experimental sets.

The electrophysiological evaluation of nerve conduction in mice was performed after completing the behavioral tests. Sural nerve sensory conduction velocity and amplitude were measured as shown in Figure 2A. In the case of the sensory sural nerve, the amplitude of the response was unaffected by paclitaxel treatment (Fig. 2B). The sensory conduction velocity was calculated in 2 ways, considering the latency to both the onset and the peak of the response (Figs. 2C and D, respectively). The onset reflects the conduction velocity of large-diameter fibers, while the peak is mainly determined by the latency in the response of the small-diameter fibers. The administration of paclitaxel in mice demonstrated a significant decrease of the sensory conduction velocity of the sural nerve (Figs. 2C and D, respectively). Figure 3A shows the paradigm used to measure amplitude and the velocity of the sensory nerve conduction of caudal nerve. In the caudal nerve sensory conduction experiment, both the velocity and the amplitude (number of fibers conducting the electrical stimulus) of the response resulted slightly, but significantly, decreased in paclitaxel-treated animals (Figs. 3B and C, respectively). DDD-028 was able to counteract the alteration of nerve signal transmission caused by paclitaxel. In particular, after the administration of DDD-028, the sensory nerve conduction velocities of both the sural (hind limb; Figs. 2C and D) and caudal (tail; Fig. 3B) nerves increased significantly in paclitaxel-treated mice coadministered with DDD-028. Furthermore, the amplitude of the sensory caudal nerve response was also increased in paclitaxel-treated mice receiving DDD-028, albeit this effect did not reach a statistical significance (Fig. 3C). By contrast, pregabalin was ineffective in restoring the alterations of sensory nerve signal transmission induced by paclitaxel (Figs. 2 and 3). Paclitaxel treatment did not affect the responses relative to the motor nerve conduction of sciatic nerve (hind limb; Fig. 4A) for both velocity and the amplitude of the signal (Figs. 4B and C). In addition, when we analyzed caudal nerve motor conduction (tail; Fig. 5A), we found that velocity (Fig. 5B) and amplitude (Fig. 5C) were not influenced by paclitaxel treatment nor by DDD-028 coadministration.

### 3.3. Effect of DDD-028 on paclitaxel-induced damage to peripheral nervous tissues

**Figure 6.**
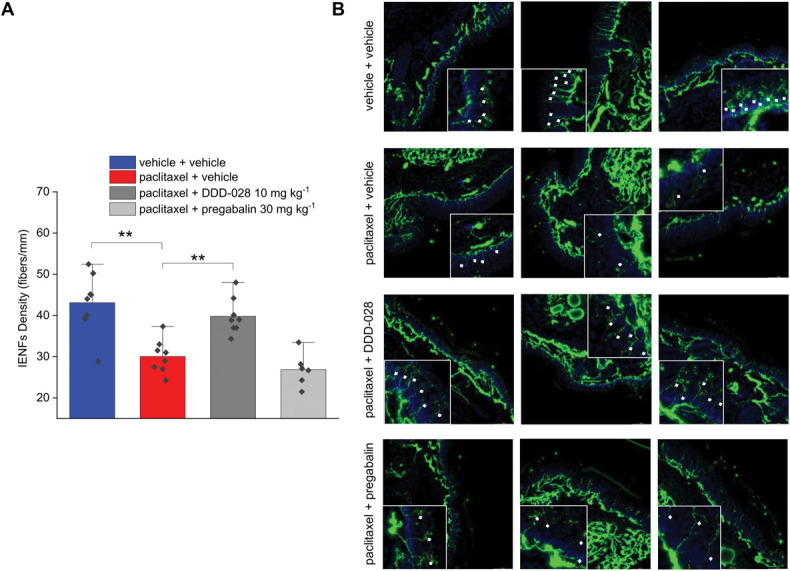
Effect of DDD-028 on intraepidermal nerve fibers loss caused by paclitaxel injection in mice. Paclitaxel 2.0 mg kg^−1^ was dissolved in a mixture of 10% Cremophor EL and saline solution and intraperitoneally (i.p.) administered on 4 alternate days (1, 3, 5, and 8). DDD-028 (10 mg kg^−1^) and pregabalin (30 mg kg^−1^) were suspended in 1% carboxymethylcellulose (CMC) sodium salt and daily administered per os starting on the first day of paclitaxel treatment and continuing 1 week after the end of treatment (from day 1 to day 17). Animals were killed on day 18, 24 h after the last treatment. Quantitative analysis of IENF density (fibers/mm) was performed by collecting 6 independent fields (through a 40× objective) in the skin of each animal and counting the number of single PGP9.5-positive fibers crossing the epidermis–dermis boundary (basal membrane). (A) Intraepidermal nerve fiber (IENF) density (fibers/mm) and (B) representative photomicrograph of PGP9.5-positive intraepidermal nerve fibers (white arrows) in mice paw skin. Each value represents the mean ± S.E.M of 6 to 8 mice per group performed in 2 different experimental sets. **P < 0.01.

The administration of paclitaxel in mice resulted in a significant reduction of the IENF density as indicated by the number of PGP9.5-positive fibers/mm of skin (Fig. 6A) and as emerged from the illustrations (Fig. 6B). DDD-028 was able to effectively counteract the loss of IENF caused by paclitaxel. Indeed, the number of nerve fibers crossing the epidermis increased significantly in the group of animals receiving DDD-028 (10 mg kg^−1^) compared with the paclitaxel group. By contrast, the loss of IENF observed after paclitaxel administration in mice was not altered by pregabalin (30 mg kg^−1^) treatment (Figs. 6A and B).

**Figure 7.**
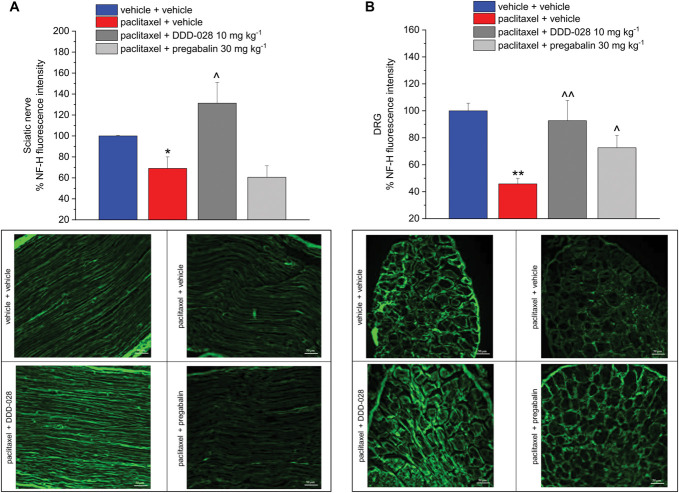
Effect of DDD-028 on NF-H expression in the sciatic nerve and dorsal root ganglia (DRG) of paclitaxel-treated mice. Paclitaxel 2.0 mg kg^−1^ was dissolved in a mixture of 10% Cremophor EL and saline solution and intraperitoneally (i.p.) administered on 4 alternate days (1, 3, 5, and 8). DDD-028 (10 mg kg^−1^) and pregabalin (30 mg kg^−1^) were suspended in 1% carboxymethylcellulose (CMC) sodium salt and daily administered per os starting on the first day of paclitaxel treatment and continuing 1 week after the end of treatment (from day 1 to day 17). Animals were killed on day 18, 24 h after the last treatment. Immunofluorescence intensity was measured by automatic thresholding algorithm and expressed in percentage in comparison to the vehicle + vehicle group set as 100%. Analyses were performed on at least 5 different images for each animal, collected through a 40× objective. (A) Neurofilament heavy-chain (NF-H) fluorescence intensity and representative photomicrograph of NF-H-positive fibers (green) in mice sciatic nerve. (B) Neurofilament heavy-chain (NF-H) fluorescence intensity and representative photomicrograph of NF-H-positive fibers (green) in mice DRG. Each value represents the mean ± S.E.M of 6 to 8 mice performed in 2 different experimental sets. *P<0.05 and **P<0.01 vs vehicle + vehicle group; ^*P*<0.05 and ^^*P*<0.01 vs paclitaxel + vehicle group.

Paclitaxel also induced a significant reduction of the neurofilament heavy-chain (NF-H) expression in the sciatic nerve, as measured by percent decrease of fluorescence intensity in comparison to the vehicle control group (histogram and representative images in Fig. 7A). The NF-H level was significantly reduced also in the DRG from paclitaxel-treated mice with respect to controls, as illustrated by the graph and the images (Fig. 7B). In both sciatic nerve and DRG tissues, repeated daily administration of DDD-028 was able to prevent the decrease in the expression of NF-H caused by paclitaxel. Pregabalin partially counteracted NF-H loss in the DRG (Fig. 7A) but not in the sciatic nerve (Fig. 7B). Moreover, paclitaxel treatment caused a derangement of the nerve fibers' disposition in the sciatic nerve, which was restored by DDD-028 treatment as shown in Figure 7A. Finally, the plasma level of neurofilament light chain (NF-L), a direct marker of neurotoxicity, was significantly increased in paclitaxel-treated mice with respect to controls (Table 1). Consistent with the protective profile described above, the plasma NF-L concentration in DDD-028-treated animals displayed values similar to those of the vehicle group. Again, by contrast, the NF-L level in pregabalin-treated animals was not statistically significant from the paclitaxel group.

**Table 1:** Neurofilament light-chain (NF-L) levels in the plasma.

Treatment	NF-L (pg/mL)
Vehicle + vehicle	12.4 ± 1.2
Paclitaxel + vehicle	45.8 ± 3.6*
Paclitaxel + DDD-028 10 mg kg^−1^	23.4 ± 1.6^
Paclitaxel + pregabalin 30 mg kg^−1^	38.7 ± 2.9

Neurofilament light-chain concentration in plasma: effect of DDD-028. Paclitaxel 2.0 mg kg21 was dissolved in a mixture of 10% Cremophor EL and saline solution and intraperitoneally (i.p.) administered on 4 alternate days (1, 3, 5, and 8). DDD-028 (10 mg kg21) and pregabalin (30 mg kg21) were suspended in 1% carboxymethylcellulose (CMC) sodium salt and daily per os administered starting on the first day of paclitaxel treatment and continuing 1 week after the end of treatment (from day 1 to day 17). On day 18, after behavioural assessments, blood was collected by exsanguination (from vena cava). Plasma was obtained by centrifugation, and NF-L was dosed in triplicate by the ELISA method. Each value represents the mean 6 S.E.M of 8 mice performed in 2 different experimental sets. *P ,0.05 vs vehicle + vehicle; ^∧^P , 0.05 vs paclitaxel + vehicle.

### 3.4. Effect of DDD-028 on paclitaxel-induced dorsal root ganglia and sciatic nerve structural alterations

**Figure 8.**
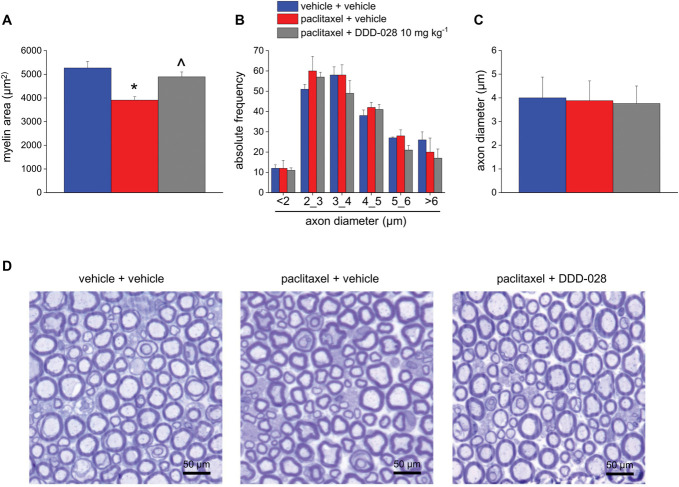
Effect of DDD-028 on morphology and morphometry of sciatic nerve of paclitaxel-treated mice. Paclitaxel 2.0 mg kg^−1^ was dissolved in a mixture of 10% Cremophor EL and saline solution and intraperitoneally (i.p.) administered on 4 alternate days (1, 3, 5, and 8). DDD-028 (10 mg kg^−1^) was suspended in 1% carboxymethylcellulose (CMC) sodium salt and daily administered per os starting on the first day of paclitaxel treatment and continuing 1 week after the end of treatment (from day 1 to day 17). Animals were killed on day 18, 24 h after the last treatment. Sciatic nerves were fixed in 2.5% glutaraldehyde in cacodylate buffer, pH 7.4, for 24 hours. Tissues were embedded in epoxy resin, and sections of 0.8 μm thickness were cut using an ultramicrotome. Each section was then stained with toluidine blue. In the sciatic nerve, myelin area (A), number of fibers separated by axon diameter (B), and axon diameters (C) were assessed and used as parameters to evaluate alterations in sciatic nerve morphology and morphometry. (D) Representative photomicrograph of mice sciatic nerve stained with toluidine blue. *P < 0.05 vs vehicle + vehicle; ^*P* < 0.05 vs paclitaxel + vehicle.

**Figure 9.**
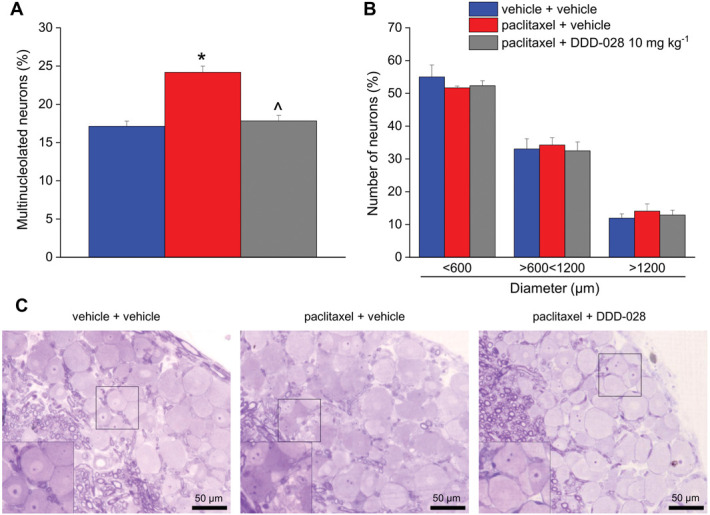
Effect of DDD-028 on morphology and morphometry of DRG of paclitaxel-treated mice. Paclitaxel 2.0 mg kg^−1^ was dissolved in a mixture of 10% Cremophor EL and saline solution and intraperitoneally (i.p.) administered on 4 alternate days (1, 3, 5, and 8). DDD-028 (10 mg kg^−1^) was suspended in 1% carboxymethylcellulose (CMC) sodium salt and daily administered per os starting on the first day of paclitaxel treatment and continuing 1 week after the end of treatment (from day 1 to day 17). Animals were killed on day 18, 24 h after the last treatment. DRG were fixed in 2.5% glutaraldehyde in cacodylate buffer, pH 7.4, for 24 hours. Tissues were embedded in epoxy resin; sections (0.8 μm) were cut using an ultramicrotome and stained with toluidine blue. DRG were studied by measuring the number of neurons showing multinucleolated neurons as a sign of toxic alteration (A); the soma area (B) was also assessed by stratifying neurons in 3 sizes: small (<600 µm diameter), medium (>600 < 1200 µm diameter), and large (>1200 µm diameter). (C) Representative photomicrograph of mice DRG stained with toluidine blue. *P < 0.05 vs vehicle + vehicle; ^*P* < 0.05 vs paclitaxel + vehicle.

Morphometric evaluations of nerve fiber diameter, axon diameter, and myelin thickness were performed using osmium-fixed nerves (Fig. 8). Paclitaxel was able to cause structural damage to sciatic nerve, as indicated by the decreased area covered by myelin in paclitaxel-treated animals compared with the vehicle control, which was significantly counteracted by the repeated administrations with DDD-028 (Fig. 8A). On the other hand, nerve fiber and axon diameters were not affected by either the chemotherapeutic treatment or the DDD-028 administration (Figs. 8B and C). Overall, paclitaxel-induced slight alterations in the morphology of the sciatic nerve tissue were prevented by DDD-028 as shown in Figure 8D. Dorsal root ganglion neurons from paclitaxel-treated animals showed a significantly increased number of nuclei with eccentric nucleoli and multinucleolated neurons compared with the vehicle control (Fig. 9A). No significant alterations in the diameter of DRG neurons were detected among the experimental groups (Fig. 9B). The administration of DDD-028 exerted a protective effect also on DRG because from the histological analysis a significant reduction of multinucleolated neurons was detected (Figs. 9B and C).

## 4. Discussion

**Figure 10.**
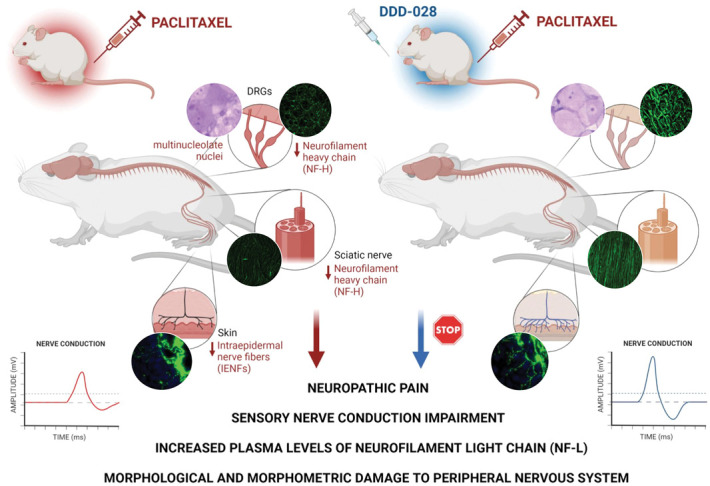
Graphical abstract.

The results obtained in the present work demonstrated the neuroprotective efficacy of DDD-028 against paclitaxel-induced neuropathy. The repeated treatment with DDD-028 was able to restore a near-normal sensory nerve conduction in paclitaxel-treated animals, in addition to counteracting the painful symptomatology. The restoration of peripheral nerve functionality was associated with the capacity of DDD-028 to protect sensory neurons from paclitaxel-induced damage, as demonstrated by the prevention from IENF loss, the normalization of neurofilament levels, and the preservation of the integrity of both sciatic nerve and DRG morphology. The broad spectrum of beneficial effects of DDD-028 is schematized in Figure 10.

Patients with CIN develop predominantly sensory disorders which entail both negative symptoms, such as hypoesthesia and hypoalgesia, and positive symptoms, such as paresthesia, temperature sensitivity, allodynia, and pain.^87^ Negative symptoms are commonly linked to the loss of larger myelinated fibers, whereas positive and pain symptoms are often associated with damage to the smaller (unmyelinated) fibers.^40,82^

In our previous study,^61^ we observed that DDD-028 was highly effective against paclitaxel-induced neuropathic pain in rats. The present work provides further direct evidence of neuroprotective properties of DDD-028 against the damage caused by paclitaxel to the peripheral nervous system. From a functional point of view, to confirm the presence of sensory distal neuropathy, patients undergo nerve conduction studies.^3,4,43,47,69^ In line with previous evidence,^13,54,74,83^ mice subjected to paclitaxel treatment developed significant alterations in sensory nerve conduction amplitude and velocity, without relevant motor conduction impairments, similarly to what was observed in patients. Sensory nerve action potential amplitude correlates with sensory thresholds, and in the case of subdermal electrodes, results correlate with sensory signs and distal pressure-induced evoked nerve potentials in sensory neuropathy.^46,86^ It is worth to note that significantly higher doses of paclitaxel and intravenous route of administration have been used in several works performing electrophysiological analysis on nerves in rodents.^16,21,39,54,70,90,91^ Considering that nerve conduction parameters are dose-dependently affected, and that the neuropathy worsens by increasing the dose of the chemotherapeutic agent, it is not surprising that the effect observed in these previous works might be more enhanced than the one observed by us. We used a relative low dose of chemotherapy compared with those used in the previous works to be as close as possible to paclitaxel dosage used in patients. Indeed, the dose of 2.0 mg kg^−1^ was sufficient to induce a persistent neuropathy in mice, as observed from both behavioral and electrophysiological evaluation. Electrophysiology study showed a slight but significant decrease of both the sural and the caudal sensory nerve conduction velocity after paclitaxel treatment, which is consistent with previous works where an analogue dose of paclitaxel has been used.^12,13,22^ The protective effects of DDD-028 on both nerve conduction impairments and pain associated with CIN are not linearly correlated^11^ but clearly reflect the strong neuroprotective efficacy of DDD-028, which has been confirmed by the observation of an umbrella-like effect in counteracting the wide range of manifestations of chemotherapy-induced polyneuropathy at both histological and molecular levels.

Paclitaxel-induced peripheral neuropathy in mice has found to be associated with a significant reduction in IENF density in skin biopsy,^10^ which is considered the gold standard for diagnosing small-fibre pathology at the clinical level.^23,52,53^ Notably, different anticancer treatments result in a loss of nerve fibers that innervate the *epidermis*, which can manifest in a time-dependent manner even 6 months after treatment has been terminated.^8,14,49^ The IENFs are particularly important in transmitting noxious mechanical and thermal information,^65^ and their loss represents a common feature of a wide range of chronic painful neuropathic conditions,^42,67,72,84^ including paclitaxel-induced neuropathy.^13^ Studies pertaining to the relationship between IENF loss and functional impairments have found that the loss of IENFs is most pronounced in areas specific to pain,^66, 72, 77^ and that decreased IENF density correlates with alterations to warmth detection and pinprick sensitivity.^84,88^ Interestingly, the loss of IENFs caused by paclitaxel treatment was found to coincide with the development of mechanical hyperalgesia.^13^ Thus, it seems reasonable that the antihyperalgesic efficacy of DDD-028 might be also related to its protective effect against the damage to IENFs caused by paclitaxel. Moreover, DDD-028 was also able to maintain the physiological levels of NF proteins in the sciatic nerve and in the plasma, which have been recently proposed as a biomarker in chemotherapy-induced polyneuropathy.^9,45,50,92^ Because chemotherapy-associated toxicity leads to a primarily axonal polyneuropathy,^15^ injured peripheral neurons release NF-L, which subsequently leads to increased blood NF-L concentrations.^9,24,40^ Here, we observed that the increase of plasma NF-L levels caused by paclitaxel in mice was significantly reduced by the administration of DDD-028. Accordingly, the immunoreactivity related to NF-H in both the sciatic nerve and the DRG of paclitaxel-treated mice was significantly increased by DDD-028 treatment, which preserved peripheral neurons, particularly their axons, from chemotherapy-associated toxicity; pregabalin was only partially effective in this regard. Deletion of NF-H has been reported to lead to atrophy of motor axons, decrease in conduction velocity, and reduced numbers of unmyelinated sensory axons.^30,48,51^ Therefore, the protective effect of DDD-028 on NF-H (nerves and DRG) may likely be linked to the improved performance observed in nerve conduction studies, as well as to the preservation of IENFs. Commonly, conduction slowing and latency prolongation correlate with peripheral nerve demyelination, while reduction in amplitude is caused by axonal loss.^15^ Interestingly, the histopathology of the sciatic nerve by toluidine blue staining showed no obvious changes in the axon number of paclitaxel-treated mice, but a significant damage to myelin sheaths was detected, as in previous findings.^64^ It is thus likely that the reduction in nerve conduction velocity associated with paclitaxel treatment was caused by a damage to myelin, which can be counteracted by coadministering DDD-028. On the other hand, DRG from paclitaxel-treated mice showed an increased number of multinucleated neurons, which may result from either self-fusion of neurons or fusion of neurons and glial cells. Even if neuronal cell–cell fusions may occur spontaneously during development and repair, most of these events have been described in the context of specific conditions or insults, which include anticancer chemotherapy, viral infection, axonal injury, as well as the presence of stem or precursor cells.^28,36^ Whether these cell–cell fusion events are beneficial or detrimental to the neurons involved remains unresolved. Nevertheless, mechanisms do exist to prevent uncontrolled cell–cell fusion, thereby maintaining neurons as individual units,^36^ suggesting again the neuroprotective potential of DDD-028, which prevented neuronal cells fusion induced by paclitaxel treatment in the DRG.

In our previous study we observed that DDD-028-mediated acute pain relief is dependent on a positive modulation of nicotinic system.^61^ As observed for the pain-relieving mechanisms, the positive modulation of α7 nAChR subtype may also mediate the neuroprotective effects of DDD-028.^6,17,68^ Moreover, the α7 nAChRs are involved in the modulation of synaptic transmission in peripheral neurons, due to their presence on nerve terminals^2^ and their activation promotes the neuroprotective functions of astrocytes against oxaliplatin neurotoxicity.^29^ In this regard, it is interesting to note that DDD-028 suppressed the pathological over activation of glia caused by paclitaxel in rats.^61^ The same compound might also exert antioxidant effects by the activation of α7 nAChRs, which are involved in the molecular pathway of Nrf2.^18,37^ In support of this hypothesis, previous ex vivo analysis showed that DDD-028 was able to reduce the oxidative damage to DRG.^61^ Further mechanistic studies into the neuroprotective profile of DDD-028 are in progress to elucidate the precise downstream pathway.

It is important to stress that available options for the pharmacological treatment of CIN are still limited to few drugs, such as gabapentinoids (gabapentin and pregabalin) and duloxetine. Although the last guidelines of ASCO recommend the use of duloxetine for treatment of CIN,^38,56^ the most commonly dispensed drug after initiating neurotoxic chemotherapy was found to be gabapentin^34^ and its congener pregabalin.^57^ It is also important to consider that, according to the aforementioned ASCO guidelines, no agent on the market can be used with confidence as a neuroprotective strategy against CIN in the general setting. The only options still available in daily practice therefore remain treatment modification and treatment withdrawal.^38,56^ We demonstrated that DDD-028 is more effective than pregabalin in counteracting the disease. Unlike the treatment with pregabalin, which can only be considered just a symptomatic intervention, DDD-028 showed a strong neuroprotective profile, counteracting the damage to the nervous system and the establishment of conduction abnormalities caused by paclitaxel in mice.

The evidence presented herein demonstrated clear advantages of using DDD-028 for the management of CIN over pregabalin and suggests that DDD-028 should be investigated further as both a prophylactic and interventional approach in patients undergoing therapies with taxanes or other neurotoxic drugs.

## Conflict of interest statement

The authors have no conflicts of interest to declare.
